# Protocetraric and Salazinic Acids as Potential Inhibitors of SARS-CoV-2 3CL Protease: Biochemical, Cytotoxic, and Computational Characterization of Depsidones as Slow-Binding Inactivators

**DOI:** 10.3390/ph15060714

**Published:** 2022-06-04

**Authors:** Lorenza Fagnani, Lisaurora Nazzicone, Pierangelo Bellio, Nicola Franceschini, Donatella Tondi, Andrea Verri, Sabrina Petricca, Roberto Iorio, Gianfranco Amicosante, Mariagrazia Perilli, Giuseppe Celenza

**Affiliations:** 1 Department of Biotechnological and Applied Clinical Sciences, University of L’Aquila, Via Vetoio 1, 67100 L’Aquila, Italy; lorenza.fagnani@graduate.univaq.it (L.F.); lisaurora.nazzicone@graduate.univaq.it (L.N.); nicola.franceschini@univaq.it (N.F.); sabrina.petricca@univaq.it (S.P.); roberto.iorio@univaq.it (R.I.); gianfranco.amicosante@univaq.it (G.A.); mariagrazia.perilli@univaq.it (M.P.); giuseppe.celenza@univaq.it (G.C.); 2 Department of Life Sciences, University of Modena and Reggio Emilia, Via Campi 103, 41125 Modena, Italy; 224762@studenti.unimore.it

**Keywords:** cysteine enzyme, SARS-CoV-2 3CL protease, lichen secondary metabolites, inhibition assay, slow binding inhibitor, inactivator

## Abstract

The study investigated the inhibitory activity of protocetraric and salazinic acids against SARS-CoV-2 3CL^pro^. The kinetic parameters were determined by microtiter plate-reading fluorimeter using a fluorogenic substrate. The cytotoxic activity was tested on murine Sertoli TM4 cells. In silico analysis was performed to ascertain the nature of the binding with the 3CL^pro^. The compounds are slow-binding inactivators of 3CL^pro^ with a *K_i_* of 3.95 μM and 3.77 μM for protocetraric and salazinic acid, respectively, and inhibitory efficiency *k_inact_*/*K_i_* at about 3 × 10^−5^ s^−1^µM^−1^. The mechanism of inhibition shows that both compounds act as competitive inhibitors with the formation of a stable covalent adduct. The viability assay on epithelial cells revealed that none of them shows cytotoxicity up to 80 μM, which is well below the *K_i_* values. By molecular modelling, we predicted that the catalytic Cys145 makes a nucleophilic attack on the carbonyl carbon of the cyclic ester common to both inhibitors, forming a stably acyl-enzyme complex. The computational and kinetic analyses confirm the formation of a stable acyl-enzyme complex with 3CL^pro^. The results obtained enrich the knowledge of the already numerous biological activities exhibited by lichen secondary metabolites, paving the way for developing promising scaffolds for the design of cysteine enzyme inhibitors.

## 1. Introduction

The novel coronavirus SARS-CoV-2 belongs to the β-genus of the *Coronaviridae* family, which includes SARS-CoV and MERS-CoV and other approximately 26 species, classified according to their genome sequences and serological reactions [1].

Coronaviruses are characterized by an envelope and the largest known positive-stranded RNA viral genome. [2]. SARS-CoVs genomes encompass 26 to 32 kilo-nucleotides, comprising a variable number of open reading frames (ORFs) [3]. The SARS-CoV-2 genome is organized in 14 ORF encoding for structural, non-structural, and accessory 27 proteins [4]. ORF1ab and ORF1a encode for overlapping replicases, pp1ab (790 kDa) and pp1a (486 kDa), which mediate viral replication and transcription [5].

These non-structural proteins are processed by two cysteine proteases encoded by ORF1a, the PL^pro^ papain-like protease, and the 3CL^pro^ main protease. PL^pro^ cuts at the first three cleavage sites of its polyprotein, while 3CL^pro^ cleaves at the remaining 11 sites, resulting in the release of 15 non-structural proteins (nsp) [6]. PL^pro^ and 3CL^pro^ play the same role in SARS- and MERS-CoVs [7].

The 3CL^pro^ is essential for viral replication and is highly conserved among 3CLs. SARS-CoV-2 3CL^pro^ is organized in three domains: domain I (residues 8–101), domain II (residues 102–184), and domain III (residues 201–303). The main protease is active as a homodimer but exists in a monomer–dimer equilibrium in solutions [7,8,9,10,11]. The homodimer contains two protomers, each of which has a Cys-His dyad located in the active site between domains I and II. The protein structure is mainly characterized by β-sheets (domains I-II), while the dimer interface is composed of adjacent helices (domain III) and loops [12,13]. In the active site, a hydrogen-bond interaction between Ser1 (the N-terminal residue) of a protomer and residue Glu166 from another protomer is essential for keeping the active pocket in the correct shape and conformation for efficient catalysis.

Faced with the looming need to curb the advance of SARS-CoV-2 infection, strategies were to develop vaccines to prevent and drugs to treat the infection. Among the possible viral targets, a significant body of work has been focused on inhibitors of the main protease. For instance, Pfizer’s oral antiviral drug Paxlovid™ appears to drastically reduce the risk of hospitalization or death [14,15].

Most investigations aim to repurpose approved drugs [16,17,18] or natural compounds [19,20]. The pharmaceutical potential of lichens has received great attention [21] considering their numerous biological properties: anti-inflammatory, antimicrobial, analgesic, antipyretic, antioxidant, and antiproliferative [22,23,24,25,26,27]. Nevertheless, their therapeutic potential still needs to be fully exploited [26,28].

Depsidones are lichen secondary metabolites biosynthesized from two or more hydroxybenzoic acids [29]. This class of compounds has shown antimicrobial activity against Gram-positive and Gram-negative bacteria and fungi, cytotoxic activity against several neoplastic cell lines, and in vitro and in vivo antioxidant properties [30,31,32]. It has also been demonstrated that some depsidones can interact with the SOS response system in human pathogenic bacteria by inhibiting bacterial recombinase RecA [30,33].

**Figure 1:**
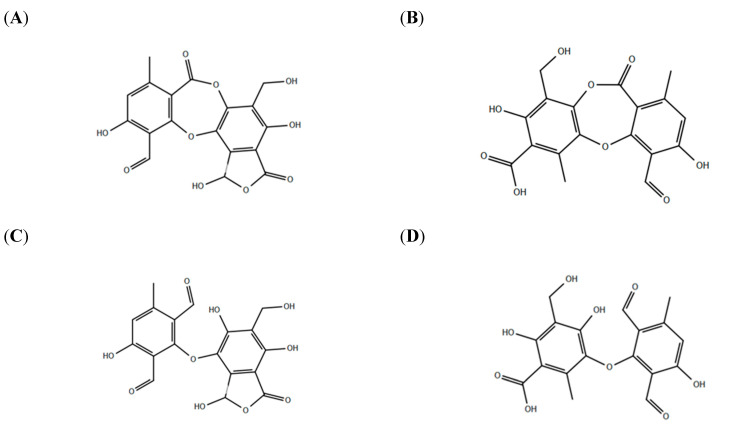
Molecule structures. (**A**) Salazinic acid and (**B**) protocetraric acid: the cyclic ester conformers (closed) docked against SARS-CoV-2 main protease are represented. (**C**) Salazinic acid and (**D**) protocetraric acid: the open conformers docked against SARS-CoV-2 3CL protease are represented. All the molecules were drawn using 2D sketcher tool of Maestro (Maestro, Schrödinger, LLC, New York, NY, USA).

This study investigated the potential inhibitory activity of salazinic and protocetraric acids (Figure 1), two depsidones, against SARS-CoV-2 3CL^pro^.

## 2. Results

### 2.1. Determination of the Kinetic Parameters

**Figure 2:**
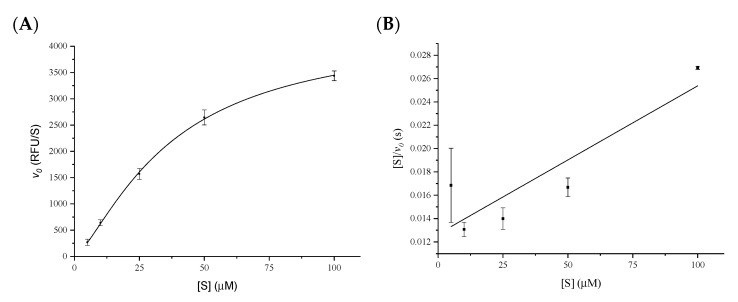
Determination of the kinetic parameters of 3CL protease. (**A**) Plot of the non-linear fit of the initial rates versus five different substrate concentrations fitting the Hill equation for cooperative enzymes. (**B**) Hanes–Woolf linearization of the initial rates versus several substrate concentrations in order to verify the non-Michaelian behaviour of the enzyme. In this case, the coefficient of determination R^2^ of the interpolated line was equal to 0.7260, which excludes the Michaelian model.

The plot of the initial rate (v_0_) versus the substrate concentration for the 3CLpro enzyme is well described by the Hill equation for a cooperative enzyme (Figure 2A). The V_max_ value was 4457 ± 290 RFU s^−1^, corresponding to 0.0235 µM s^−1^ with a *k_cat_* value of 2.73 s^−1^. The enzyme’s affinity for the substrate, K, was calculated as 31.5 ± 3.8 µM and the Hill coefficient h was equal to 1.43 ± 0.14. The non-Michaelian behaviour of the enzyme was confirmed by the Hanes–Woolf linearization of the Michaelis–Menten equation (Figure 2B). Any deviation from the hyperbolic kinetic can be easily determined by looking at the coefficient of determination R^2^ of the interpolated line, which was equal to 0.7260.

### 2.2. Inhibition Assay

**Figure 3:**
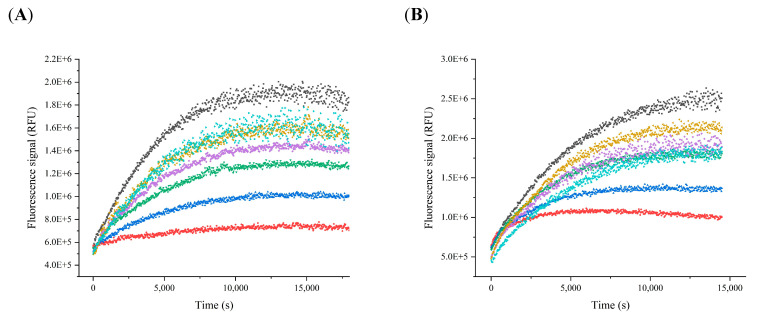
Time course at various inhibitor concentrations. (**A**) Protocetraric acid; (**B**) salazinic acid. Black, positive control; light blue, 5 µM; yellow, 10 µM; magenta, 25 µM; green, 50 µM; blue, 75 µM; red, 100 µM. RFU, relative fluorescence unit.

The time-course plots at inhibitor concentrations ranging from 5 µM to 100 µM are consistent with a slow-binding inactivator for protocetraric acid (Figure 3A) and salazinic acid (Figure 3B).

**Figure 4:**
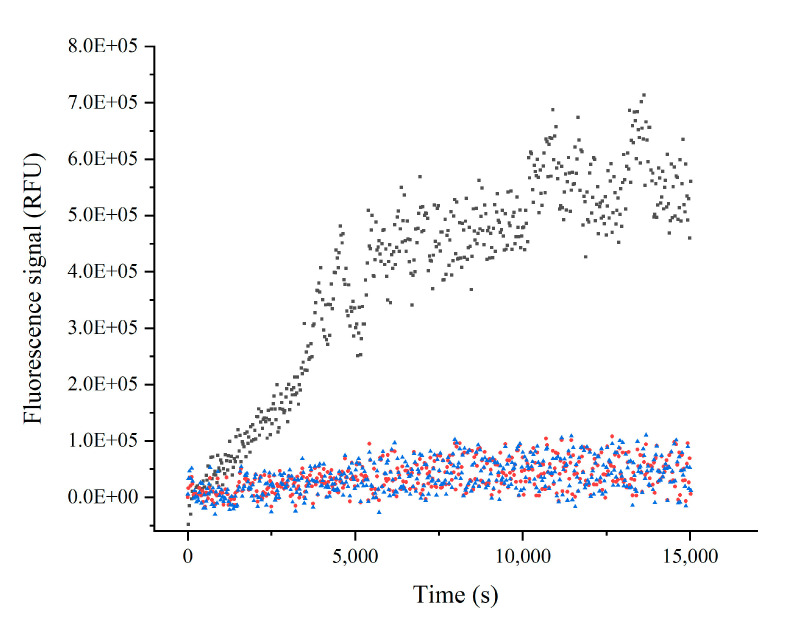
The irreversibility of the inhibition was ascertained through the recovery of the enzymatic activity. Black, positive control; red, protocetraric acid 50 µM; blue, salazinic acid 50 µM.

The irreversibility of the inhibition was determined by measuring the recovery of the enzymatic activity after a large dilution of the enzyme-inhibitor complex at 50 µM of each inhibitor. The plot in Figure 4 shows that it was not possible to observe a recovery of the enzymatic activity after the dilution of the inhibitors; this is a diagnostic of the formation of a stable enzyme–inhibitor complex, which ultimately allows inhibitors to be defined as inactivators. The described mode of action of the inhibitors conforms to the reactions reported in reaction scheme where the initial EI complex, characterized by *k_on_* and *k_off_* rate constants, is slowly followed by the formation of a stable EI* adduct, characterized by an inactivation rate constant, *k_inact_*.

To overcome the substrate depletion effect, the time-course curves at different inhibitor concentrations were fitted to Equation (3). *K_i_^app^* and *k_inact_* values were estimated by analyzing the entire set of data. The results from this analysis are reported in Table 1. Both compounds behave in the same way with almost equal *K_i_^app^* and *k_inact_* values.

**Figure 5:**
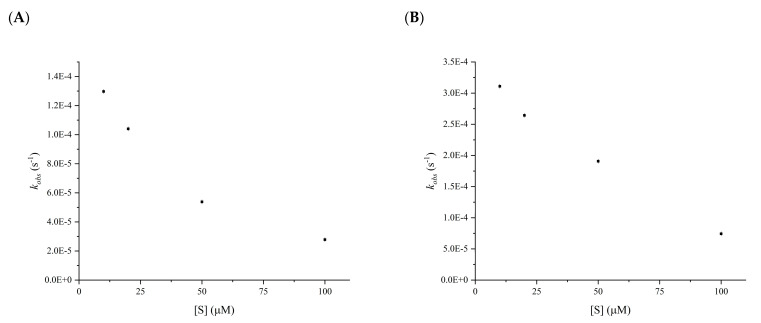
Plot of the *k_obs_* versus the substrate reporter, ranging from 10 µM to 100 µM, at a concentration of inhibitors at 75 µM for the determination of the mechanism of inhibition. (**A**) protocetraric acid, (**B**) salazinic acid.

The last issue to be resolved concerns the mechanism of inhibition. It was ascertained by calculating the *k_obs_* values from *K_i_^app^* and *k_inact_* determined at a fixed concentration of the inhibitor, 75 µM for both compounds, varying the concentration of the reporter substrate. As shown in Figure 5, both compounds acted competitively with the substrate since the *k_obs_* value decreases as the substrate concentration increases. The determination of a competitive mechanism of inhibition allowed the determination of the real *K_i_^app^* value by applying Equation (6). The *K_i_^app^* values for both compounds were lower than 4 µM (Table 1).

### 2.3. Molecular Docking

A preparative docking was performed only for cyclic esters conformers in extra precision mode (XP mode, non-covalent docking) to confirm the ability of the ligands to complement the active site of 3CL^pro^. Docking predicted that the ligands could fit appropriately in the active site establishing many interactions with the surrounding residues (data not shown).

**Figure 6:**
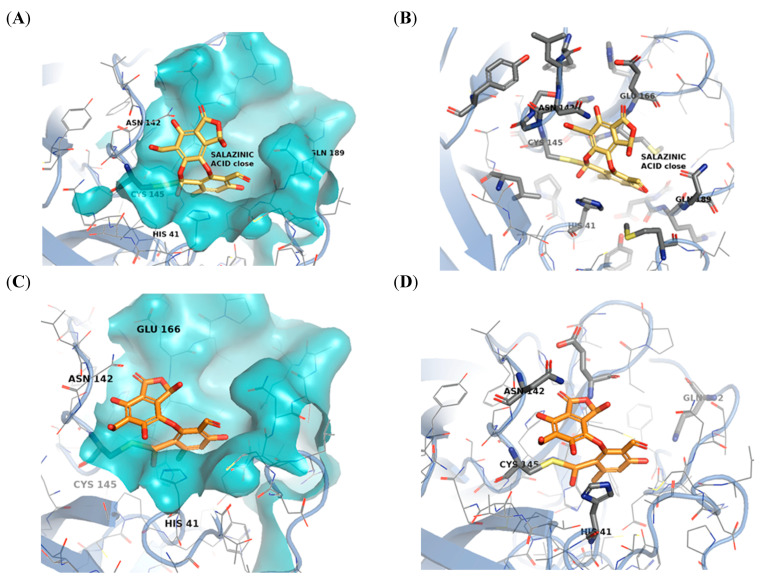
(**A**,**B**) Cyclic ester salazinic acid covalently bound to SARS-CoV-2 3CL^pro^. (**C**,**D**) Open salazinic acid conformer covalently bound to CoV-2. Cys145, His41, and Glu166, residues essential for the enzymatic activity of 3CL^pro^, are highlighted. All figures were prepared with PyMOL (PyMOL Molecular Graphics System, Version 2.5 Schrödinger, LLC).

**Figure 7:**
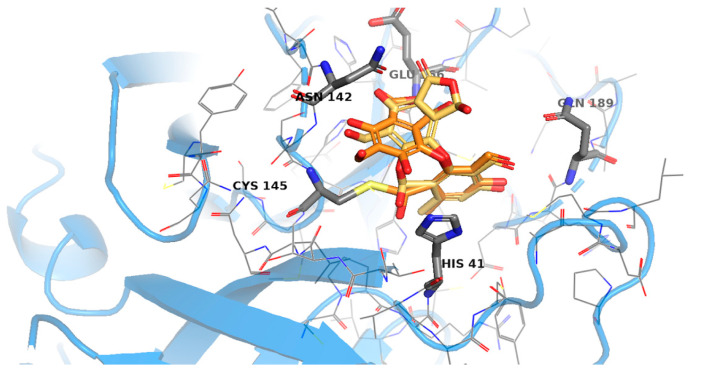
Comparison between open (orange) and closed (dark yellow) conformation for salazinic. The benzofuranone ring rearranges and makes a hydrogen bond with Glu166. Figures were prepared with PyMOL (PyMOL Molecular Graphics System, Version 2.5 Schrödinger, LLC).

**Figure 8:**
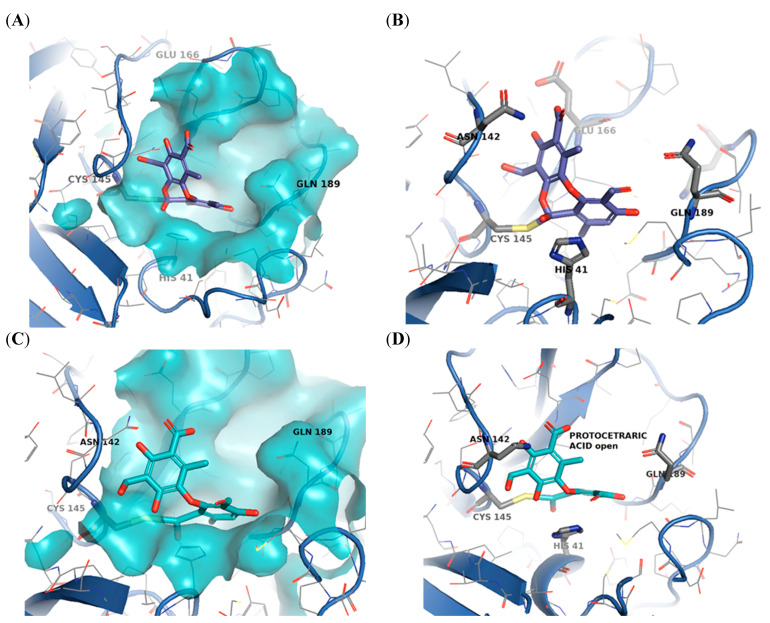
(**A**,**B**) Cyclic ester protocetraric acid covalently bound to SARS-CoV-2 3CL^pro^. (**C**,**D**) Open protocetraric acid conformer covalently bound to SARS-CoV-2 3CL^pro^. Cys145, His41, and Glu166, residues essential for the enzymatic activity of 3CLpro, are highlighted. All figures were prepared with PyMOL (PyMOL Molecular Graphics System, Version 2.5 Schrödinger, LLC).

Then, covalent docking (CovDOCK) was carried out for salazinic and protocetraric acid, both in open and closed conformations (Figure 1). The docking procedure depicts the formation of a covalent interaction, in agreement with kinetics analysis findings. The docking results reveal how both ligands adjusted well in the active site. For the predicted enzyme-inhibitor complex, binding is guided by the covalent bond with Cys145, the reactive residue in the active site, and was further stabilized by specific interactions such as hydrogen bonds, salt bridge, and π–π interactions (Figure 6, Figure 7 and Figure 8).

What is noteworthy is the nucleophilic attack mediated by the Cys145 involves ligands at their cyclic ester, one of the possible but not exclusive reactive points in the molecules.

More in detail, the binding prediction for salazinic acid as a cyclic ester (closed conformer) (Figure 6A,B) confirmed that catalytic Cys145 establishes a covalent bond with the ester carbon carbonyl. The aromatic ring forms a π-π stacking with His41, and the hydroxyl group of the adjacent ring interacts by H-bond with Gly143 and Leu141 (Figure S3, Supplementary File).

In the binding prediction for salazinic acid carrying the hydrolysed ester (open conformer) (Figure 6C,D), the covalent attack by Cys145 takes place as mentioned, and the molecule takes advantage of the higher conformational freedom due to its open form. As a result, the molecule can tune some established interactions with the surrounding active site residues as it slightly rearranges in the binding site. The aromatic side ring maintains the π-π stacking with His41, which also forms a salt bridge interaction with the bicycle hydroxyl. Dihydro benzofuranone hydroxyl forms two H-bond interactions with Ser144 and Gly143. Moreover, the furanone establishes an additional H-bond with Glu166 (Figure S4, Supplementary File).

The binding energy of the salazinic acid as cyclic ester is consistent (docking score −5.982). After its rearrangement in the active site allowing a better orientation of the bulky benzo furanone rings, the open conformation is energetically more favorited (docking score −7.007). Moreover, the number of allowed docking poses from closed to open conformations increases remarkably (52 with respect to 82) (Figure 7).

In the binding prediction for protocetraric acid as a cyclic ester (closed conformer) (Figure 8A,B), as already seen for salazinic acid, the carbonyl carbon of the cyclic ester undergoes a nucleophilic attack by catalytic Cys145 and is covalently bonded to it. The side aromatic ring forms a π-π stacking with His41. The aromatic carboxylic acid is involved in an H-bond with Asn142. The ligand seems to be stabilized further in the active site, mainly by apolar interactions (Figure S5, Supplementary File).

The open protocetraric acid conformer is predicted to undergo a covalent attack by Cys145 as well; moreover, the cleaved cyclic ester slightly rearranges in the binding site thanks to the gained conformational freedom, as also observed for the open salazinic acid conformer (Figure 8C,D). As a matter of fact, the molecule loses the bond with Asn143, but it establishes additional interactions with the surrounding active site residues: the aromatic hydroxyl methyl group interacts by H-bonds with Gly143, while the aromatic carboxylic group establishes an H-bond with Glu166. The aromatic ring maintains its π-π stacking with His41, which is also involved in a hydrogen bond with the vicinal carbonyl group carried by the ligand (Figure S6, Supplementary File). Few hydrophobic residues in the proximity further stabilize the complex. The improvement in the binding is confirmed by increasing the docking score and the number of allowed orientations for each conformer.

### 2.4. Cell Proliferation and Viability in TM4 Epithelial Cells

The trypan-blue exclusion assay did not reveal any cytotoxic effects induced by protocetraric acid and salazinic acid on TM4 cells at the tested doses, ranging from 1 to 80 µM, up to 48 h exposure (data not shown). It could not be possible to explore higher concentrations since the poor solubility of the compounds can increase the toxic effect of DMSO.

## 3. Discussion

The World’s population has faced an invisible enemy two times, from 2002 to 2019, who silently stole lives and shocked the lives of those who survived it. In late 2002, a previously unknown HCoV, called Severe Acute Respiratory Syndrome (SARS), emerged in Guangdong, the People’s Republic of China, and rapidly spread in Asia and Canada. The spread of the virus was contained early in 2003, but more than 8000 cases, including 800 deaths, were reported during this period [34,35]. December 2019 was characterized by a coming and going of discordant news about a new type of flu, determined by a novel type of coronavirus. The first cases of this pathology, called coronavirus disease 2019 (Covid-2019), have been reported in Wuhan, Hubei Province [36], and rapidly led to a global pandemic [37,38]. To date, after more than two years, we still remain in a pandemic state.

Despite the recent advances to control the current outbreak, the rapid spread of SARS-CoV-2 is still challenging to face. Although research has turned to the development of effective vaccines, the repurposing of approved drugs and natural compounds is turning out to be the most promising approach for combating the virus infection. Among the possible viral targets, a significant body of work has been focused on 3CL main protease inhibitors.

Motivated by the effects and consequences of COVID-19 spread, we addressed our investigations on the potential inhibitory activity of lichen secondary metabolites on SARS-CoV-2 3CL^pro^. Lichens produce a wide variety of secondary metabolites with most being synthesized by the mycobiont [27]. Approximately 1050 secondary compounds have been identified [30].

In this study, we investigated protocetraric acid and salazinic acid (Figure 1), two compounds belonging to the class of depsidones, some of the most abundant lichen secondary metabolites [30]. Depsidones are biosynthesized from depsides, which are themselves formed by the condensation of two or more hydroxybenzoic acids where the carboxyl group of one molecule is esterified with a phenolic hydroxyl group of a second molecule [29].

As demonstrated by the kinetic study, protocetraric and salazinic acids act as time-dependent inactivators. The slow binding and time-dependent modes of action have been previously described in other 3CL^pro^ inhibitors. Fu L. et al. demonstrated that Boceprevir and GC376 are time-dependent inhibitors that covalently bind the catalytic cysteine of SARS-CoV-2 3CL^pro^. GC376 is more potent than Boceprevir, with an *IC_50_* value of 0.15 µM versus 8.0 µM [39]. Although due to the competition effect of the substrate, the *IC_50_* values cannot be directly correlated with the inhibition constants, and the *K_i_* values estimated for protocetraric acid and salazinic acid are presumably of the same order of magnitude as those estimated for Boceprevir and GC376. However, it must be considered that the best way to express the “power” of an inactivator is to refer to the *k_inact_*/*K_i_* ratio, which represents the inactivation efficiency. For instance, although protocetraric and salazinic acids have different inhibition constants, their *k_inact_*/*K_i_* ratios are comparable (Table 1). Both lichen compounds show a behavior that is not new in the scenario of viral protease inhibitors. For instance, Boceprevir, a serine protease inhibitor used to treat HCV since 2011 covalently binds the NS3 protease [18], whereas GC376 is a covalent inhibitor of cysteine proteases of picornaviruses, noroviruses, and coronaviruses [40,41].

Recently, the oral antiviral candidate Paxlovid™, developed by Pfizer Inc., raises new hopes of COVID-19 recovery [42,43]. It is formulated as a combination of two oral drugs: PF-07321332 and ritonavir. The first, also known as nirmatrelvir, inhibits the SARS-CoV-2-3CL protease. The second drug, ritonavir, is used only to slow nirmatrelvir metabolism [14,44,45]. As is the case of Boceprevir, GC376 and the tested lichen compounds, nirmatrelvir covalently binds catalytic Cys145 to form a reversible thioimidate adduct, as determined by the 1.6-Å crystal structure in complex with SARS-CoV-2 3CL^pro^ [43].

In this study, the data achieved through the kinetic study were further confirmed by molecular docking. Specifically, the binding orientations of salazinic and protocetraric acids, here described as covalent and irreversible inactivators of SARS-CoV-2 protease, were predicted. To achieve the reported results, which replicate the mechanism of action of our inhibitors, each ligand was docked as a cyclic ester (closed conformation) and as a hydrolyzed ester (open conformation).

As a matter of fact, we were able to draw in silico the reaction coordinates: the ligand oriented itself as a cyclic ester (closed conformation, data not shown) in the active site, proximal to catalytic Cys145, corresponding to the formation of the non-covalent EI complex (reaction scheme). Subsequently, the ligand, still as a closed conformer, undergoes a nucleophilic attack by the sulfur of the catalytic Cys145 at the carbonyl carbon of the cyclic ester: an intermediate with high energy is formed.

As shown by the in silico study, the formation of the open conformers in the enzyme’s active site leads to energetically favored ligand rearrangements, which represent the driving force behind the decay of the high-energy transition intermediate to a more stable EI* complex (reaction scheme).

The molecules have numerous possible sites susceptible to nucleophilic attack by Cys145. From a pharmacological standpoint, the most plausible site is the carbonyl carbon of the ester bond of the central cyclic ester, which is present in both molecules, and the carbonyl carbon of the hydroxyfuranone ring, which is present only in salazinic acid and not in protocetraric acid.

Notably, the nucleophilic attack by catalytic Cys145 on both salazinic and protocetraric acids involves the cyclic ester, even though the inhibitors are decorated with several other reactive electrophilic centers. Specifically, the results of the kinetics of inhibition and the observed behavior of the two molecules ruled out the hydroxyfuranone ring as a possible pharmacophore. This hypothesis is also confirmed by the results of the in silico study, where the central cyclic ester is reported as the preferred site of the cysteine attack.

The results obtained from the in silico study are consistent with the co-crystallization studies performed by other authors on protease 3CL inhibitors of SARS-CoV-2 [46,47,48,49]. These studies show that residues in the catalytic pocket that interact with protocetraric acid and salazinic acid are involved in binding other inhibitors. Specifically, His41, Gly143, and Glu166 may play a fundamental role in stabilizing the inhibitor within the catalytic pocket.

We can summarize that by looking at the results from the in silico study and the kinetic behavior, where the molecules were characterized as irreversible inactivators of the enzyme, it is reasonable to assume the formation of a covalent bond between the inhibitors and the catalytic cysteine.

This study also investigated the potential adverse effects of protocetraric and salazinic acids on TM4 cells, a well-established model for testing drug cytotoxicity [50,51]. Previous evidence showed that both compounds have antiproliferative effects on a variety of tumoral cellular models, including colorectal and melanoma cancer cell lines. In contrast, no adverse effects were detected on normal cell culture [52,53,54,55,56]. In line with this, our results confirmed the absence of any significant cytotoxic effect for both compounds up to 48 h of exposure at the tested doses.

Finally, there is one aspect of this study that the authors feel should be highlighted. Some of the recent studies on 3CL protease inhibition present methodological issues. The first of these problems concerns the non-Michaelian behavior of the enzyme. For example, in many studies, the cooperative behavior, known since 2006 for the equivalent enzyme in SARS-CoV [57,58,59,60], is not diagnosed, making the analysis of the inhibition data impossible when a Michaelian approach is attempted [16].

The second aspect concerns the management of slow inactivators that exhibit substrate depletion, as in the case of Boceprevir and GC376 [39]. The approach to the problem proposed in this study resolves these critical issues, allowing the evaluation of kinetic parameters of inhibition, even in a non-Michaelian enzyme, and for slow-binding inhibitors.

## 4. Materials and Methods

### 4.1. Reagents

SARS-CoV-2 3CL Protease was purchased from Sigma-Aldrich (https://www.sigmaaldrich.com/italy, accessed on 15 May 2022). (3CL Protease Assay Buffer, 3CL Protease Substrate, and 3CL inhibitor GC376 were purchased from Vinci Biochem (Firenze, Italy). Dimethyl sulfoxide (DMSO) and dithiothreitol (DTT) were purchased from Sigma-Aldrich Chemical Co. (Saint Louis, MO, USA). Ultrafree-MC microcentrifuge filters with a pore size of 5000 Da were purchased from Sigma-Aldrich (https://www.sigmaaldrich.com/italy, accessed on 15 May 2022). Dulbecco’s modified Eagle medium (DMEM)/Ham’s F-12 50/50 Mix (with 2 mM L-glutamine and 15 mM Hepes) was purchased from Corning Life Sciences (Manassas, VA, USA). Fetal bovine serum (FBS), and horse serum (HS) were from EuroClone (Pero, MI, Italy). Penicillin-streptomycin was from TermoFisher Scientific (Carlsband, CA, USA). Trypan-blue assay and other reagents were from Sigma-Aldrich Chemical Co. (Saint Louis, MOi, USA). Protocetraric acid and salazinic acid (Figure 1A,B) were isolated from lichens collected in various Chilean regions, including the Chilean Antarctic Territory, and were donated by Prof. Marisa Piovano (Universidad Técnica Federico Santa María, Valparaíso, Chile)

### 4.2. Spectrofluorimetric Assay

SARS-CoV-2 3CL protease activity assay was performed, as suggested by the manufacturer with some modifications, by measuring the fluorescence intensity in a microtiter plate-reading fluorimeter using the fluorogenic substrate DABCYL-KTSAVLQSGFRKME-EDANS.

Reactions were performed in a black 96-well microplate using a final volume of 100 μL in each well. Experiments were carried out in the reaction buffer supplied by the manufacturer in the presence of dithiothreitol (DTT) to preserve the enzyme integrity in these long-run experiments. DTT was added to 3CL Protease Assay Buffer before use, with a final concentration of 1 mM.

3CL protease was used at the final concentration of 89 nM. The enzyme was preincubated with the inhibitor under examination for 30 min at room temperature in a 96-well microplate. The reaction was started by adding the 3CL substrate solution to each well at a concentration value of 10 μM.

The fluorescence intensities in each well were evaluated with the spectrofluorimetric assay by Beckman Coulter DTX-880 Multimode Detector Microplate Reader. The excitation and emission wavelengths were 360 nm and 535 nm, respectively. The percentage of inhibition was calculated as the fractional residual activity of 3CL protease incubated 30 min with lichen compounds. Additionally, the 3CL inhibitor GC376 was used at a concentration of 100 µM as a positive control and the inhibitor buffer as inhibitor control.

A standard curve was generated to convert the relative fluorescence unit (RFU) to the amount of the cleaved substrate. The curve has been obtained by inducing the complete hydrolysis of the substrate at concentrations ranging from 5 to 100 µM (Figure S1, Supplementary File).

### 4.3. Determination of Kinetic Parameters

The kinetic parameters of 3CL SARS-CoV-2 protease were determined by incubating the enzyme as previously described. The experiment was performed at several substrate concentrations, ranging from 5 µM to 100 µM, by measuring the initial rates (*v_0_*, RFU/s) of the progress curves that were calculated at less than 10% hydrolysis of the initial substrate concentration. The plot was well described by the non-linear fitting of the Hill equation (Figure 2A):

(1) $$v_{0}=\frac{V_{max}\;{\left[S\right]}^{h}}{K^{h}+{\left[S\right]}^{h}}$$

where *h* is the Hill coefficient, and *K* is related to substrate occupancy at one site on the substrate affinity of the other site. Any deviation from the Michaelis–Menten equation, where *h* is equal to 1, can be easily visualized by the Hanes–Woolf linearization method, where the initial rate *v_0_* is plotted as [*S*]/*v_0_* against [*S*]: when *h* is equal to 1, and the plot is represented by a straight line with a coefficient of determination *R^2^* of the interpolated line close to 1, while for *h* ≠ 1, *R^2^* is far from 1 (Figure 2B).

### 4.4. Inhibition Assays

The inhibition assays were conducted at six inhibitor concentrations (Figure 3), ranging from 5 µM to 100 µM. The reactions were performed in a black 96-well microplate as previously described.

The irreversibility of the inhibition was ascertained through the recovery of the enzymatic activity after a 100-fold dilution of the enzyme-inhibitor complex at 50 µM of each inhibitor by microcentrifuge tube filters.

Briefly, the reaction was performed as previously described (spectrofluorimetric assay). After a large dilution of the enzyme-inhibitor complex, it was centrifuged at 5000× *g* with a temperature of 4 °C for about an hour and a half. The retentate was retrieved and after the addition of substrate at 10 µM, the recovery of the enzymatic activity was measured (Figure 4).

Once the irreversibility was ascertained, it was possible to identify the reaction scheme. In this scheme of reaction, two kinetic parameters can describe enzyme inactivators: the constant of inhibition, *K_i_*, and the constant rate of inactivation *k_inact_*, as well as the *k_inact_*/*K_i_* ratio. *K_i_* and *k_inact_* are traditionally determined by the simplified version of the well-known Morrison–Walsh equation for slow-binding inhibitors (Equation (2)) [61]:

$$\begin{array}{cc} E+I\begin{array}{c} k_{on}\\ ⇄\\ k_{off} \end{array}EI\begin{array}{c} k_{inact}\\ \to \end{array}EI^{*} & \mathrm{Reaction}\;\mathrm{scheme} \end{array}$$

(2) $$\left[P\right]=\frac{v_{i}\;\left(1-e^{k_{obs}t}\right)}{k_{obs}}$$

where *v_i_* is the initial rate of the progress curve, and *k_obs_* is the rate of interconversion between the initial rate vi and the steady-state *v_ss_*, which is equal to zero in the case of an inactivator.

As previously described, 3CL^pro^ exhibits a cooperative behavior and, thus, is not ascribed to a Michaelian enzyme model. Since the Hill equation is empirical, it is not possible to derive equations describing the enzyme’s behavior in the presence of inhibitors. Using Michaelian models to analyze experimental data would lead to distortions in the results and their misinterpretation. It must also be considered that an essential assumption for the application of Equation (2) is that the substrate concentration does not change significantly during the time course. Unfortunately, the very slow binding nature of the inhibitors leads to a consistent depletion of the substrate during the inhibition assays. To overcome substrate depletion, Waley, in 1993, proposed an approach for slow-binding reversible inhibitors [62], which can be easily adapted to irreversible inhibitors:

(3) $$\left[P\right]={\left[S\right]}_{0}+\left[1-e^{-\delta \left(1-e^{-k_{obs}t}\right)}\right]$$

where the following is the case.

(4) $$\delta =\frac{k_{cat}{\left[E\right]}_{0}\;K_{i}}{K\;k_{inact}{\left[I\right]}_{0}}$$

(5) $$k_{obs}=\frac{k_{inact}{\left[I\right]}_{0}}{K_{i}^{app}+{\left[I\right]}_{0}}$$

One of the main problems in the application of Equation (3) lies in that the *K* (*K_m_* for Michaelian enzymes) and *k_cat_* values of the enzyme contained in the δ factor must be known. In order to overcome the problem of the slow nature of the inactivators, we globally analyzed the entire data set at different inhibitor concentrations to obtain the individual values of *K_i_^app^* and *k_inact_* by fixing the concentration of the reporter substrate at 10 µM, approximately the minimum concentration where the behavior of the enzyme can be approximated to a Michaelian model.

### 4.5. Determination of the Mechanism of Inhibition

The mechanism of inhibition for salazinic and protocetraric acids was ascertained by fixing the concentration of inhibitors at 75 µM and changing the substrate reporter concentration ranging from 5 µM to 100 µM. The rate constant *k_obs_* for each substrate concentration was derived by calculating the *K_i_^app^* and *k^inact^* values at each substrate concentration using Equation (5) and plotting the derived *k_obs_* against the substrate concentration (Figure 5). In competitive inhibitors, *k_obs_* values are expected to decrease as the concentration of the substrate increases. The real value of *K_i_* can be obtained for competitive inhibitors by Equation (6). Inhibition assays were performed in triplicates.

(6) $$K_{i}^{app}=K_{i}\left(1+\frac{{\left[S\right]}_{0}}{K}\right)$$

### 4.6. Molecular Docking

From protein and ligand preparation, receptor grid generation to molecular docking, the modelling analysis was performed with the Maestro module of Schrodinger LLC suite, version 2021-4, Schrödinger, LLC, New York, NY, USA ((L. Schrodinger. Schrodinger Software Suite Schrödinger LLC, New York (2011)).

Inhibitors salazinic acid and protocetraric acid were drawn using 2D sketcher tool of Maestro in both cyclic ester form and open conformer, the latter reflecting the effect of the covalent bond with the Cys145 (Figure 1A,B) (Maestro, Schrödinger, LLC, New York, NY, USA). Ligprep utility in the Glide module was used to generate tautomers and isomers of screened ligand. The highest number of conformers to be generated was set to 32 (LigPrep, Schrödinger, LLC, New York, NY, USA)) [63]. The ionization states for each conformer were generated at pH 7.0 ± 2.0 with EPIK; ligands were then desalted (Epik, Schrödinger, LLC, New York, NY, USA). An OPLS3 (Optimized Potentials for Liquid Simulations) force field was used for energy minimization of each compound with default parameters. The so generated ligands library was then sent to docking using Glide software (Glide, Schrödinger, LLC, New York, NY, USA).

The protein structure CoV-2 main protease (PDB ID: 7RC1) [64,65] at a resolution of 1.63 Å was downloaded from the scientific protein data bank (www.rcsb.org, accessed on 2 February 2022) [66]. The downloaded raw protein was then prepared using the protein preparation wizard of GLIDE (Glide, Schrödinger, LLC, New York, NY, USA) [67]. Missing hydrogen atoms were added, bond orders assigned, zero bond order for disulfide bonds defined, and water molecules removed. Missing residues and side chains were added with PRIME (Prime, Schrödinger, LLC, New York, NY, USA). The H-bond network was created before energy minimization using the OPLS3 force field with a default constraint of 0.30 Å RMSD [68].

The Receptor Grid Generation wizard in Glide was used for grid generation with default parameters for partial cut-off (0.25) and scaling factor (1.0) (Schrödinger, LLC, New York, NY, USA). The receptor grid defining the active site cavity was generated by selecting the centroid of ligand GRL-0686 binding to CoV-2 main protease (PDB ID: 7RC1) [65] as the centre of the grid box. The generated grid file defining active site residues was used to dock ligand using Glide software.

#### 4.6.1. Docking Protocol Validation

To validate the designed docking protocol, a covalent docking was performed with the prepared receptor CoV-2 main protease and the ligand GRL-0686 (PDB ID: 7RC1) [64,65]. The predicted binding orientation of GRL-0686 was then compared with the orientation assumed by GRL-0686 in the X-ray ternary complex, as shown below (Figure S2, Supplementary File) RMSD between the docked pose and the crystal structure pose of GRL-0686 of 0.7866 strongly supports the designed procedure.

#### 4.6.2. Extra Precision (XP) and Covalent Docking (CovDOCK)

A library of 13 ligands, prepared from the closed and the open conformation of inhibitors salazinic and protocetraric acids, as reported in paragraph 1.1, was docked in the active site of SARS-CoV main protease using GLIDE Extra Precision XP (non-covalent docking) and CovDock modes (Schrödinger, LLC, New York, NY, USA) (Table S1, Supplementary File) [67,69].

For cyclic esters conformers, molecular docking was conducted both in extra precision mode (XP mode) and in covalent mode (CovDOCK mode) (Figure 1A). For open conformers (Figure 1B), docking was run only in the CovDOCK mode. For covalent docking, residue Cys145 was set as the active residue to bound the ligand covalently: nucleophilic addition to a double bond was selected as the reaction type. For the validation procedure and covalent docking, ‘Virtual Screening (Fast)’ was selected as docking mode in the CovDock module. The default 0.8 scaling factor (vdW) and 0.15 potential charge cut-off for XP docking were set. Post-docking analysis and visualization were executed using Pymol (PyMOL Molecular Graphics System, Version 2.5 Schrödinger) [70].

### 4.7. Cell Culture and Viability Assay

TM4 (ATCC^®^ CRL1715™) mouse Sertoli cells were obtained from the American Type Culture Collection. Cells were seeded at a density of 1 × 104 cells/cm2 and routinely maintained in DMEM/F-12 50/50 Mix supplemented with 5% HS, 2.5% FBS, 100 IU/mL penicillin, and 100 μg/mL streptomycin until they reached a confluence close to 80%. Proliferation and viability rates were assessed by a Trypan blue exclusion assay. Cells were maintained at 37 °C in a 5% CO_2_ humidified atmosphere. Cells were seeded at a density of 1 × 10^4^ cells/cm^2^ and maintained in standard conditions for 24 h before treatments. Cells were then exposed to protocetraric and salazinic acid at concentrations ranging from 1 to 80 µM for 24 and 48 h.

### 4.8. Statistics and Data Analysis

All experiments were performed in triplicate. The parameters, experimental errors, and non-linear fitting were estimated and calculated using OriginPro 8.5.1 and Microsoft Excel. The analysis to determine the kinetic inhibition parameters was performed using the software DynaFit ver 4.09.047 by Petr Kuzmic (BioKin Ltd.). Modelling analysis was performed with Maestro module of Schrodinger LLC suite, version 2021-4, Schrödinger, LLC, New York, NY, USA.

## 5. Conclusions

The results of this study add an important piece to the mosaic of the already numerous biological activities of this unique class of lichen secondary metabolites: the depsidones. Considering the results obtained from the kinetic, computational, and cytotoxicity studies, we conclude that salazinic acid and protocetraric acid can be considered suitable scaffolds for developing effective inhibitors of the cysteine enzyme 3CL^pro^ of SARS-CoV-2. Furthermore, given the importance of this class of enzymes in viral infection and other pathological processes of various aetiologias, the mechanism of action of these natural molecules could represent a paradigm for the definition of potential new inhibitors.

## Figures and Tables

**Table 1:** Estimation of inhibition parameters at 10 µM of substrate.

Compound	*k_inact_* (s^−1^)	*K_i_^app^* (µM)	*K_i_* (µM)	*k_inact_/K_i_* (s^−1^ µM^−1^)
Protocetraric acid	1.19 × 10^−4^ ± 2.1 × 10^−6^	5.22 ± 0.07	3.95	3.01 × 10^−5^
Salazinic acid	1.33 × 10^−4^ ± 3.0 × 10^−6^	4.98 ± 0.09	3.77	3.53 × 10^−5^

## Data Availability

Under request.

## Supplementary Materials

The following supporting information can be downloaded at: https://www.mdpi.com/article/10.3390/ph15060714/s1, Figure S1: The calibration curve was generated to convert the relative fluorescence unit (RFU) to the amount of the cleaved substrate; Figure S2: GRL-0686 in SARS-CoV-2 main protease active site; Figure S3: Cyclic ester salazinic acid conformer covalently bound to CoV-2; Figure S4: Open salazinic acid conformer covalently bound to CoV-2; Figure S5: Cyclic ester protocetraric acid conformer covalently bound to CoV-2; Figure S6: Open protocetraric acid conformer covalently bound to CoV-2; Table S1: A library of 13 ligands, prepared from the closed and the open conformation of inhibitors salazinic and protocetraric acids.
