# Supplementary material for: Protocetraric and Salazinic Acids as Potential Inhibitors of SARS-CoV-2 3CL Protease: Biochemical, Cytotoxic, and Computational Characterization of Depsidones as Slow-Binding Inactivators
Source: Pharmaceuticals (Basel). 2022 Jun 4;15(6):714. doi: 10.3390/ph15060714 (PMC9227325; doi:10.3390/ph15060714)
Supplement: Supplementary file 1 [file pharmaceuticals-15-00714-s001.zip › pharmaceuticals-1750783-supplementary.pdf]

Supplementary files

Title

Protocetraric and Salazinic Acids as Potential Inhibitors of SARS-CoV-2 3CL Protease: Biochemical, Cytotoxic, and Computational Characterization of Depsidones as Slow-Binding Inactivators

Authors

Lorenza Fagnani 1,†, Lisaurora Nazzicone 1,†, Pierangelo Bellio 1,*, Nicola Franceschini 1, Donatella Tondi 2,*, Andrea Verri 2, Sabrina Petricca 1, Roberto Iorio 1, Gianfranco Amicosante 1, Mariagrazia Perilli 1 and Giuseppe Celenza 1

[1] Department of Biotechnological and Applied Clinical Sciences, University of L'Aquila, Via Vetoio 1, 67100 L'Aquila, Italy

[2] Department of Life Sciences, University of Modena and Reggio Emilia, via Campi 103, Modena, 41125, Italy

* Corresponding author: pierangelo.bellio@univaq.it

† The authors equally contributed to this study

	DOCKING SCORE	INTERACTIONS	DISTANCES	POSES GAINED WITH CovDock
SALAZINIC acid 'CLOSE'	-5.982	2 H-Bond<br>1 stacking	Leu 141 2.64<br>Gly 143 2.69<br>stacking 3.76	52
SALAZINIC acid 'OPEN'	-7.007	3 H-Bond<br>1 stacking His 41<br>1 salt bridge His 41	Ser144 2.77<br>Gly 143 2.77<br>Glu166 2.01<br>stacking 3.79<br>salt bridge 4.67	82
PROTOCETRARIC acid 'CLOSE'	-5.531	1 H-Bond<br>1 stacking	Asn 142 2.21<br>stacking 3.78	37
PROTOCETRARIC acid 'OPEN'	-6.332	3 H-Bond<br>1 stacking	Gly143 1.82<br>His 41 2.41<br>Glu 166 2.11<br>stacking 3.90	74

Table S1. A library of 13 ligands, prepared from the closed and the open conformation of inhibitors salazinic and protocetraric acids, as reported in paragraph 1.1, was docked in the active site of SARS-CoV main protease using GLIDE Extra Precision XP (non-covalent docking) and CovDock modes (Schrödinger, LLC, New York, NY, USA). The number of docked conformers for each ligand, generated with LigPrep: Salazinic acid cyclic ester (closed) 4; Salazinic acid (open) 4; Protocetraric acid cyclic ester (closed) 2; Protocetraric acid (open) 3.

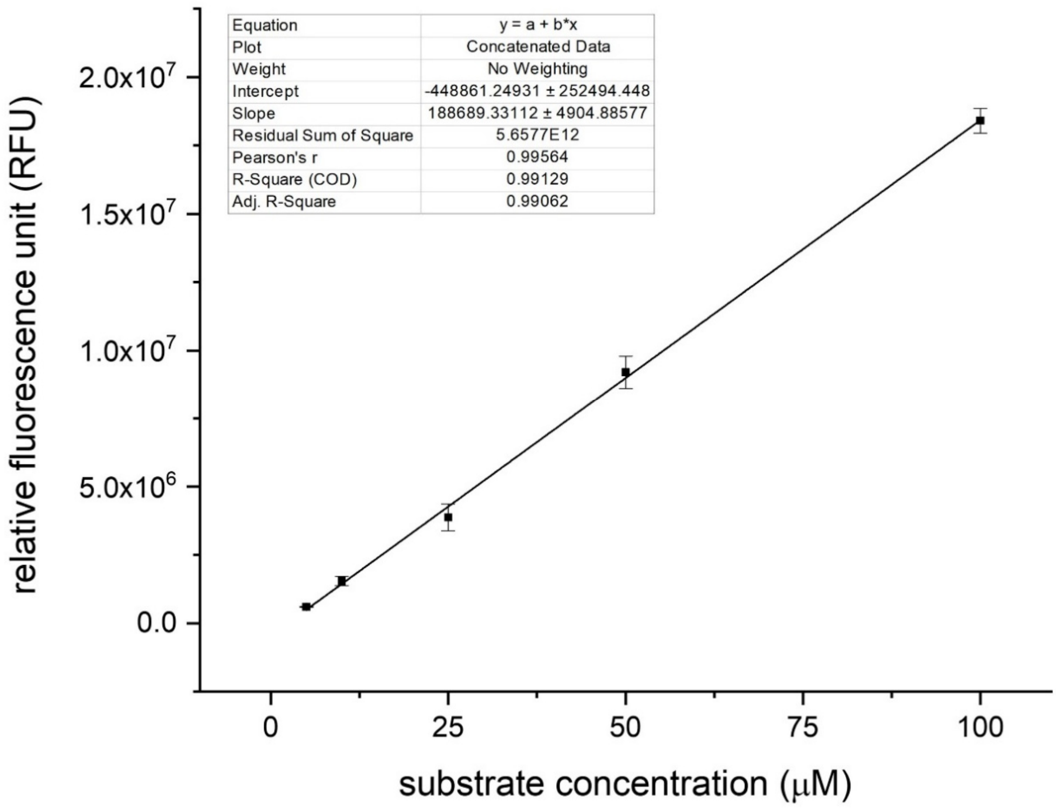

Figure S1. The calibration curve was generated to convert the relative fluorescence unit (RFU) to the amount of the cleaved substrate.

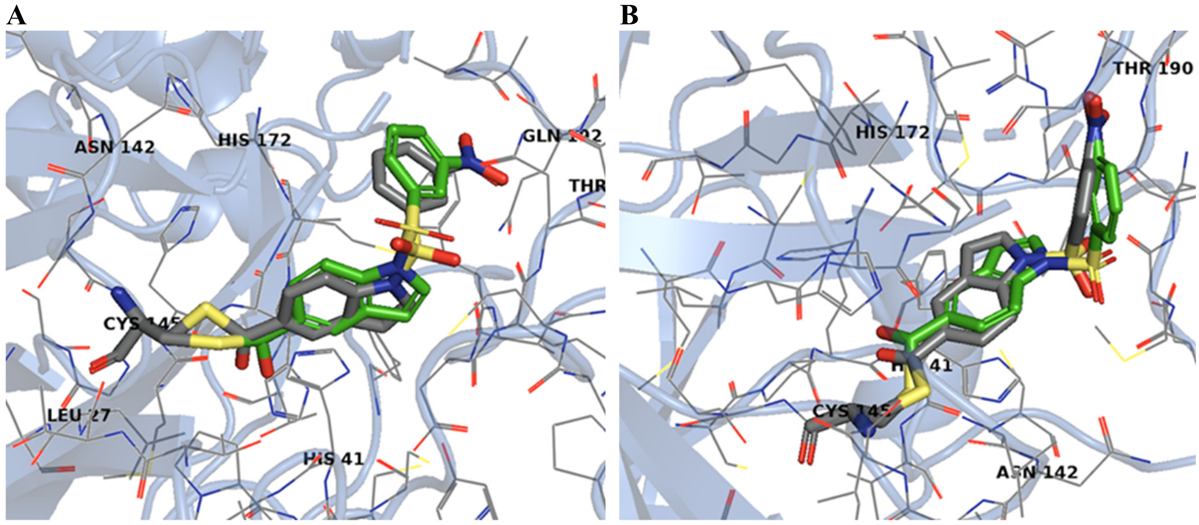

Figure S2. GRL-0686 in SARS-CoV-2 main protease active site: predicted (green) vs crystallographic orientation (gray). Figures were prepared with PyMOL (PyMOL Molecular Graphics System, Version 2.5 Schrödinger).

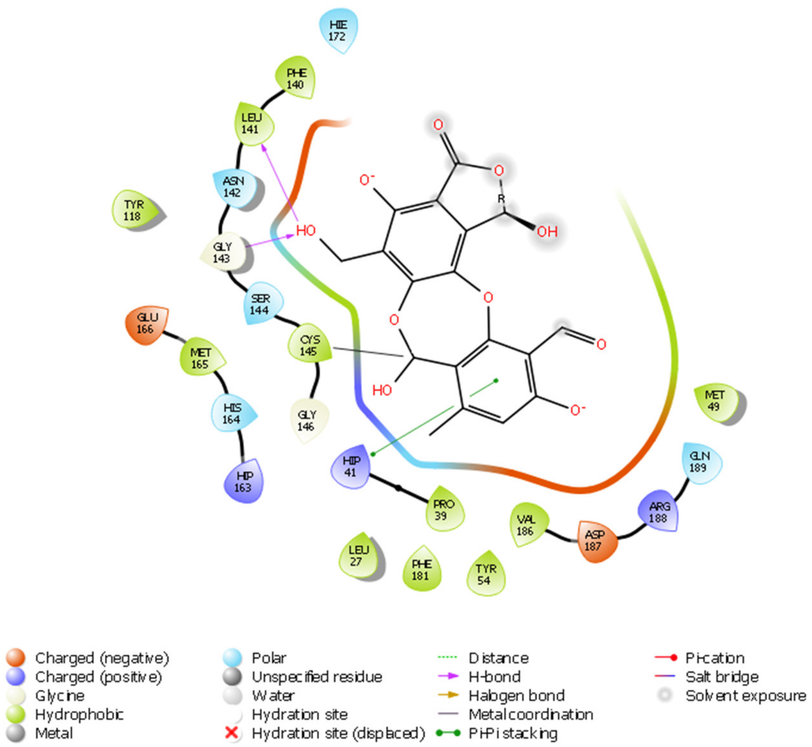

Figure S3. Cyclic ester salazinic acid conformer covalently bound to CoV-2: detailed interactions. Figures were prepared with PyMOL (PyMOL Molecular Graphics System, Version 2.5 Schrödinger, LLC).

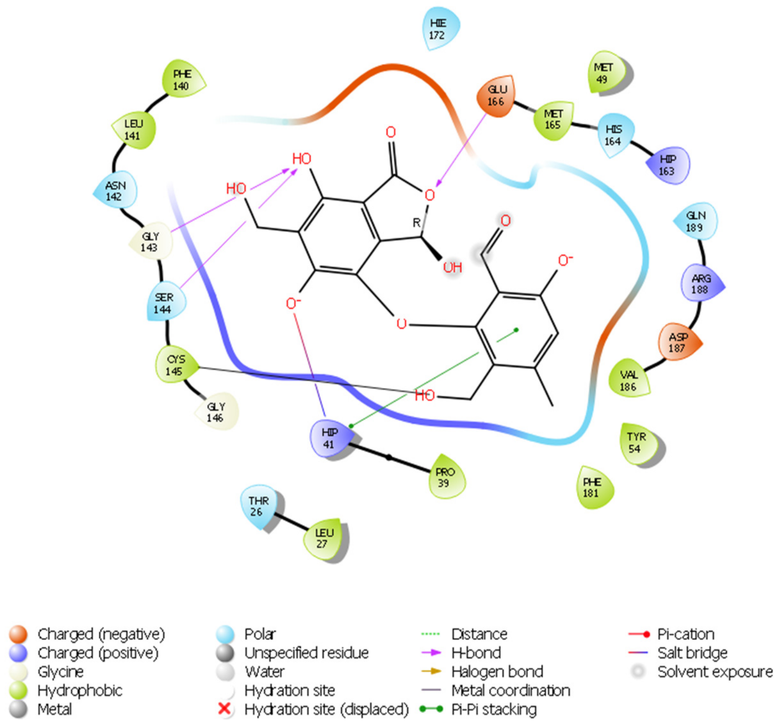

Figure S4. Open salazinic acid conformer covalently bound to CoV-2: detailed interactions. Figures were prepared with PyMOL (PyMOL Molecular Graphics System, Version 2.5 Schrödinger, LLC).

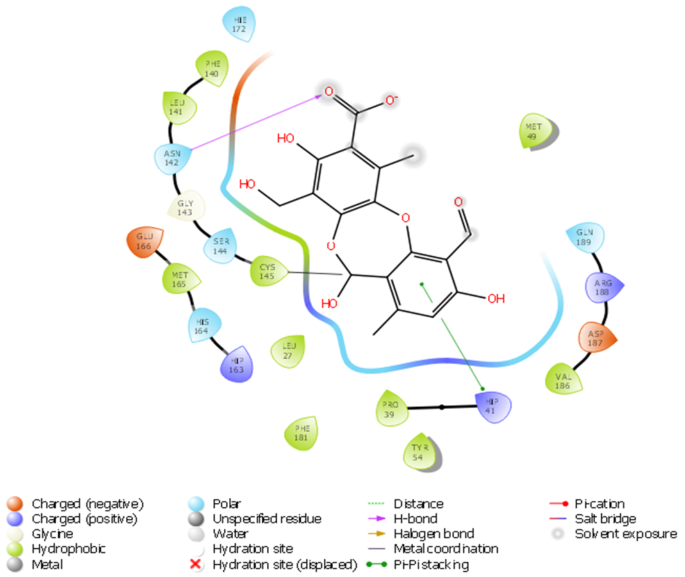

Figure S5. Cyclic ester protocetraric acid conformer covalently bound to CoV-2: detailed interactions. Figures were prepared with PyMOL (PyMOL Molecular Graphics System, Version 2.5 Schrödinger, LLC).

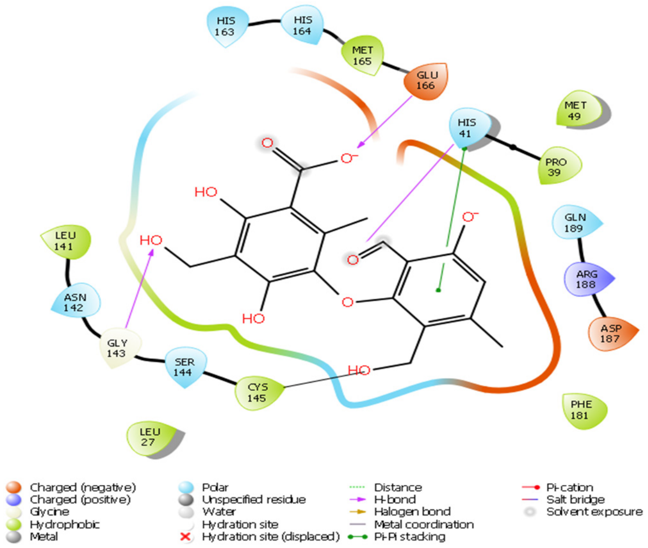

Figure S6. Open protocetraric acid conformer covalently bound to CoV-2: detailed interactions. Figures were prepared with PyMOL (PyMOL Molecular Graphics System, Version 2.5 Schrödinger, LLC).
